# Endobronchial Foreign Bodies Presenting as Intermittent Chest Pain and Productive Cough

**DOI:** 10.7759/cureus.29599

**Published:** 2022-09-26

**Authors:** Ruby Risal, Htun M Aung, Tahmina Jahir, Kamal R Subedi, Sadaf Hossain, Aye M Thida, Marie Schmidt, Danilo Enriquez

**Affiliations:** 1 Pulmonary, Interfaith Medical Center, Brooklyn, USA; 2 Medicine, Interfaith Medical Center, Brooklyn, USA; 3 Pulmonary Medicine, Interfaith Medical Center, Brooklyn, USA; 4 Internal Medicine, Interfaith Medical Center, Brooklyn, USA; 5 Internal Medicine, Jamaica Hospital Medical Center, Brooklyn, USA; 6 Medicine, Woodhull Medical and Mental Health Center, Brooklyn, USA; 7 Pulmonology, Interfaith Medical Center, Brooklyn, USA

**Keywords:** chronic cough, bronchoscopy, body stuffing, body packing, endobronchial foreign body

## Abstract

A 51-year-old male presented with intermittent chest pain for one month and productive cough with yellow sputum for seven days. He had a history of chronic kidney disease stage G3, depression, and polysubstance abuse. His chest X-ray revealed mild hazy opacity in the right lower lobe, followed by a chest computed tomography without contrast that indicated multiple nodular opacities in the left mainstem bronchus with clear lungs. The patient underwent flexible bronchoscopy where the left mainstem bronchus was found to be completely occluded by three clear plastic bags, about 1 x 0.5 cm in size containing whitish content consistent with the appearance of crack cocaine. A high index of suspicion is crucial in patients with suspected foreign body aspiration as prompt extraction of foreign bodies may prevent complications.

## Introduction

Foreign body aspiration can sometimes be life-threatening. It is less common in adults than in children. The true incidence of foreign body aspiration in adults is unknown as the cases are often misdiagnosed, may go undetected, or are discovered incidentally. It is estimated that foreign body aspiration in adults accounts for about one in 400 bronchoscopic procedures [1]. The median age of patients is 60 years, with a male-to-female ratio of 2.1-2.4:1 [2, 3]. Among those patients with a high risk of foreign body aspiration are patients with primary neurologic disorders such as seizures, brain tumors, Parkinson’s disease, mental retardation, cerebral palsy, recent cerebrovascular accidents; dental procedures; medical procedures such as tracheostomy or endotracheal tubes; trauma with associated loss of consciousness and cervicofacial injury; and alcohol or sedative use [2]. Moreover, the diagnosis is often delayed, especially in elderly patients with cognitive, neurological, or psychiatric disorders, as patients might not provide relevant information.

Depending on the anatomic structure of the tracheobronchial tree and the patient’s body posture at the time of aspiration, the site of lodgment of the foreign body may vary. Debeljak et al. reported that 67% of cases had foreign bodies in the right bronchial tree and 32% in the left [3]. In a study by Limper and Prakash, foreign body impaction was more common in the right bronchial tree than in the left and in the lower lobes than the upper [2]. The most common foreign bodies are food items such as vegetable matter, meat, and bones. Others include iatrogenic objects such as endodontic needles, tracheostomy tube segments, endotracheal tube appliances, and miscellaneous items such as straight pins, coins, and buttons.

Another rare but important differential diagnosis of aspiration in adults, especially with a history of drug abuse, is body packer or body stuffer syndrome. Body packing is an act of swallowing or inserting drug-filled packets into a body cavity, either voluntarily or through coercion [4]. Cocaine and heroin are the drugs most often involved in body packing, but methamphetamine, ecstasy, oxycodone, cannabis, and synthetic cannabinoid receptor agonists are also found in some cases. On the other hand, body stuffing involves rapid ingestion of drugs to avoid being arrested [5-7]. Therefore, people who attempt body stuffing are at increased risk for aspiration. In addition, as opposed to body packers, body stuffers do not usually plan ahead to ingest the packet, and the wrapping material used is generally prone to rupture or leakage, leading to toxicity and death.

Body packing/stuffing cases are challenging for physicians as they can lead to serious health consequences if they are not identified and treated in a timely manner. If packets rupture, sympathomimetic drugs like cocaine can cause agitation, hypertension, tachycardia, mydriatic pupils, and diaphoresis [8]. More severe toxicity can be seizures, hyperthermia, myocardial ischemia, heart failure, ventricular dysrhythmias, coma, and cardiac arrest. Sometimes they present only with mechanical complications with symptoms varying from chronic cough to acute respiratory arrest.

Patients may initially present with cough in most cases [1, 3, 9-12]. Less common symptoms may include wheezing, chest pain, hemoptysis, and recurrent pneumonia. Early complications may include acute dyspnea, asphyxia, laryngeal edema, pneumothorax, and cardiac arrest. Late complications may include bronchiectasis, hemoptysis, development of inflammatory polyps at the site of lodgment, bronchial stricture, and diminished lung perfusion on the lodgment side [2].

We describe the case of a 51-year-old male presenting to the ED in New York state with chronic intermittent chest pain, who turned out to have foreign bodies in the left main bronchus.

## Case presentation

A 51-year-old male with a history of chronic kidney disease stage G3, depression, and polysubstance abuse presented to the emergency department with intermittent chest pain for one month and productive cough with yellow sputum for seven days. He complained of intermittent left-sided chest pain, aggravated by deep inspiration, but no relieving factor. He also mentioned that his cough started as dry but progressed to be productive with yellowish sputum but not blood-stained. He denied fever, headache, body ache, shortness of breath, or wheezing. He denied taking any illicit drug recently.

On triage, the patient had a body temperature of 97.8 degrees Fahrenheit, blood pressure of 134/85 mmHg, heart rate of 98 beats per minute, respiratory rate of 18 breaths per minute, and oxygen saturation of 98% in room air. Physical examination was negative for tachypnea, tachycardia, wheezes, and crepitations.

**Figure 1:**
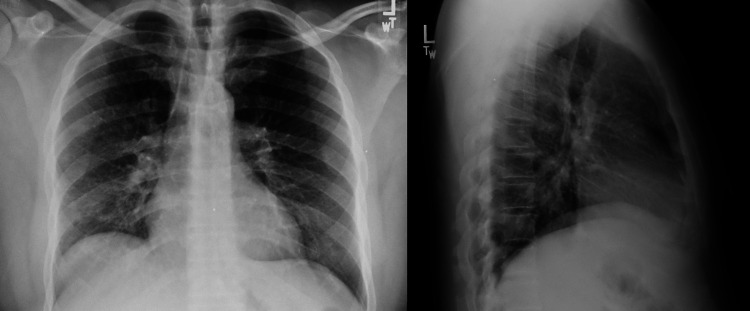
The chest X-ray showed mild hazy opacity in the right lower lobe, worrisome for early or developing pneumonia.

**Figure 2:**
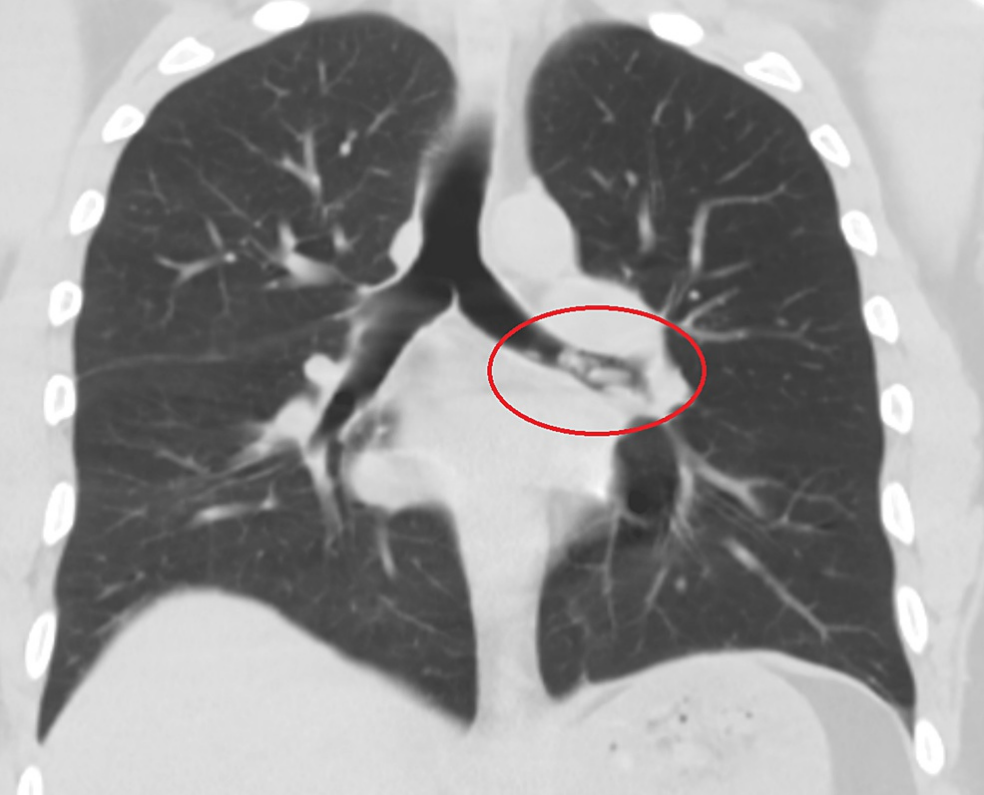
The chest computed tomography without contrast revealed multiple nodular opacities in the left mainstem bronchus with clear lungs.

**Figure 3:**
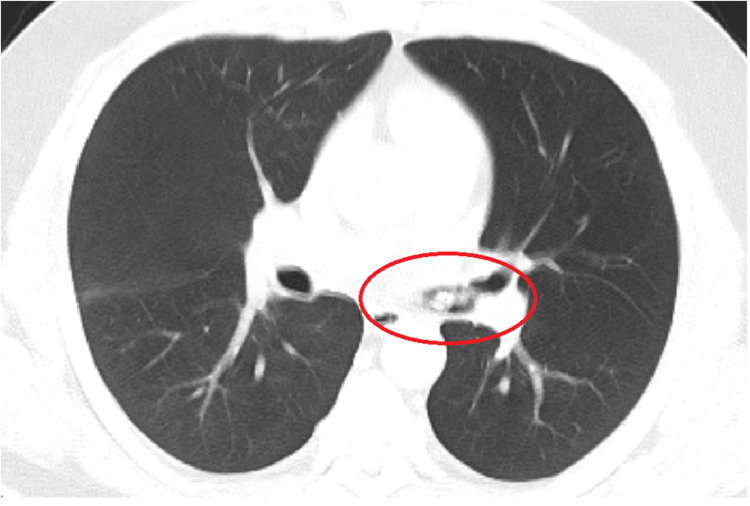
The chest computed tomography without contrast revealed multiple nodular opacities in the left mainstem bronchus with clear lungs.

Most initial laboratory investigations were within normal limits, except for elevated high-sensitivity troponin of 46.1 ng/L, which trended down to 37.3 ng/L, and urine toxicology positive for cocaine (Table 1). His electrocardiogram was normal. His chest X-ray showed mild hazy opacity in the right lower lobe, worrisome for early or developing pneumonia (Figure 1). However, his chest computed tomography without contrast revealed multiple nodular opacities in the left mainstem bronchus with clear lungs (Figures 2, 3).

**Table 1:** Initial Laboratory Investigations

Investigations	Results	Normal range
White blood cells	8.9	4.5–11 x 10^3^/µL
Hemoglobin	14.8	13.0–17.0 g/dL
Hematocrit	42.8	39–53%
Platelet count	199	130–400 x 10^3^/µL
Sodium	139	136–145 mmol/L
Potassium	3.6	3.5–5.1 mmol/L
Chloride	104	98.0–107.0 mmol/L
Carbon dioxide	23	23–31 mEq/L
Anion gap	12	6–12 mEq/L
Blood urea nitrogen	15.4	8.4–25.7 mg/dL
Creatinine	1.49	0.72–1.25 mg/dL
Glucose	152	80–115 mg/dL
Calcium	9.0	8.8–10.0 mg/dL
Magnesium	2.4	1.6–2.6 mg/dL
Phosphorus	3.6	2.3–4.7 mg/dL
Total bilirubin	0.4	0.2–1.2 mg/dL
Alkaline phosphatase	<10	10–55 U/L
Aspartate phosphatase	14	5–34 U/L
B-natriuretic peptide	<10.0	10–100 pg/mL
Lactic acid	1.0	0.5–1.9 mmol/L
High-sensitivity troponin	46.1	0.0–35.0 ng/L

**Figure 4:**
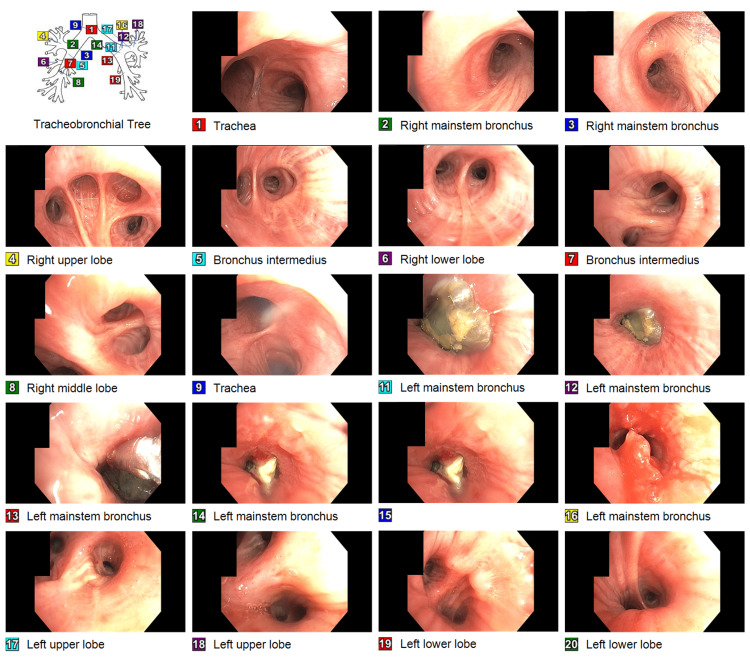
During the first bronchoscopy, the left mainstem bronchus was found to be completely occluded by three clear plastic bags, about 1 x 0.5 cm in size containing whitish content consistent with the appearance of crack cocaine. After extraction of the foreign bodies by alligator forceps, two polypoidal mucosal lesions were noted in the left mainstem bronchus. The endobronchial biopsy of one of the two mucosal lesions showed a small fragment of benign bronchial mucosa with inflammation, but no tumor was identified.

**Figure 5:**
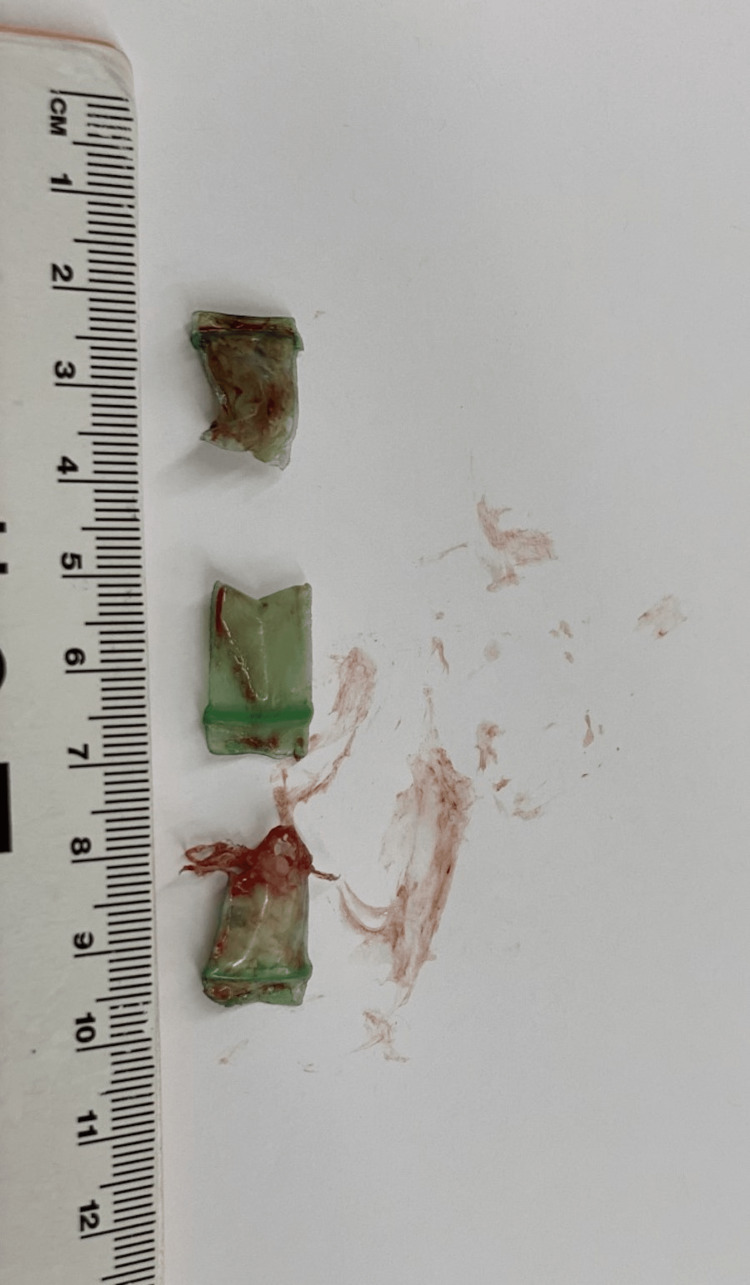
Three plastic bags, about 1 x 0.5 cm in size, containing an unknown substance, were extracted by alligator forceps from the left mainstem bronchus.

**Figure 6:**
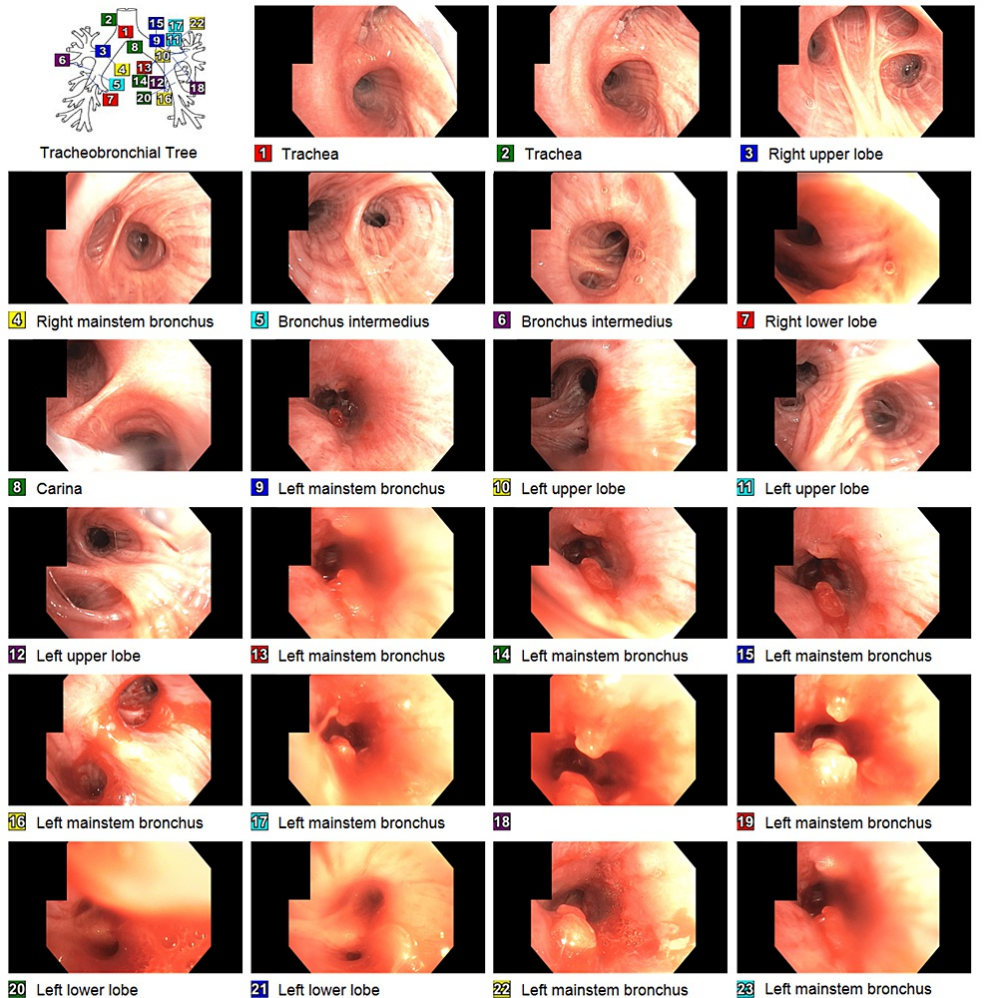
During the second bronchoscopy, the two polypoidal mucosal lesions were still noted in the left mainstem bronchus but much smaller than previous findings. The endobronchial biopsy of one of the two mucosal lesions showed benign ciliated bronchial epithelial cells with scanty mucus material.

The patient received one dose of ceftriaxone and one dose of azithromycin in the emergency department, but the antibiotics were discontinued as pneumonia was unlikely. The pulmonary team was consulted and scheduled for flexible bronchoscopy. The patient was intubated for the procedure under general anesthesia. During the bronchoscopy, the left mainstem bronchus was found completely occluded by a clear plastic bag containing an unknown substance. Alligator forceps were introduced through the bronchoscope. In a few attempts, the foreign body was grabbed, then the foreign body, alligator forceps, and bronchoscope were removed carefully as one unit to stay in the center of the airway. We maintained visualization of the foreign body throughout the procedure. The bronchoscope was passed through the endotracheal tube again and found two more similar-looking clear plastic bags about 1 x 0.5 cm in size containing whitish content consistent with the appearance of crack cocaine (Figures 4, 5). After extraction of the foreign bodies, two polypoidal mucosal lesions were noted in the left mainstem bronchus. The endobronchial biopsy of one of the two mucosal lesions showed a small fragment of benign bronchial mucosa with inflammation, but no tumor was identified. The patient underwent a second bronchoscopy three days later. During the second bronchoscopy, the two polypoidal mucosal lesions were still noted in the left mainstem bronchus but much smaller than previous findings (Figure 6). The endobronchial biopsy of one of the two mucosal lesions showed benign ciliated bronchial epithelial cells with scanty mucus material.

After the bronchoscopy, the patient mentioned that his cough and chest pain improved. As a result, the patient was discharged home with a pulmonary clinic appointment. However, the patient did not follow up with the pulmonary clinic after discharge.

## Discussion

A standard posteroanterior and lateral chest X-ray should be obtained in patients with suspected foreign body aspiration [1, 13-16]. However, it may only identify foreign bodies in 25% of cases as a minority of foreign bodies are radio-opaque, for example, coins, nails, teeth, or dental appliances. Furthermore, about 8%-80% of chest X-rays can be negative in adult tracheobronchial foreign body cases [17]. On the other hand, chest computed tomography has become the gold standard of imaging studies in patients with suspected foreign body aspiration as it is positive in 65% of cases and helps with procedural planning [1, 18, 19]. In chest computed tomography, foreign bodies generally appear as dense structures within the bronchial lumen and sometimes may present associated findings like atelectasis or hyperlucency with air trapping [19]. In case of strong suspicion and imaging remains inconclusive, chest computed tomography integrated with virtual bronchoscopy can be done to avoid bronchoscopy.

Once a foreign body is confirmed on imaging, or if a high index of suspicion is present, patients may go for bronchoscopy. Two types of bronchoscopy exist, each with its advantages and limitations.

Flexible bronchoscopy is generally considered superior to rigid bronchoscopy as an initial procedure for evaluating and managing foreign body aspiration in adult patients, especially those with smaller foreign bodies of lower airway and foreign bodies beyond the reach of rigid instruments [1]. It is widely available, can be used via endotracheal tube, allows for a more comprehensive airway survey, can be done under moderate sedation, and has a 90% overall success rate for retrieval. However, it does not provide a secure airway and cannot protect the airway from sharp objects.

Alternatively, rigid bronchoscopy is preferred in cases of acute respiratory distress as it provides a stable airway and enables bronchoscopists to manage the central airway better and asphyxiating foreign bodies [1]. It can also protect sharp, large, and more complex objects within its barrel. However, it usually requires general anesthesia, cannot visualize past the central airway, and cannot be used in patients with cervicofacial trauma.

Forceps are the most useful tool during bronchoscopy [20]. The choice of forceps depends on the size, shape, nature, and weight of the foreign body aspirated. For small organic or friable foreign bodies such as peanuts and popcorn, retrieval baskets and snares are best used [21]. Rat tooth or shark tooth grasping forceps may be preferred for flat foreign bodies like coins, dentures, and jewelry. Rubber tip grasping forceps are used for sharp or flat objects like nails, needles, pins, and blades [18]. A magnet-tip probe may also be utilized for ferrometallic objects. Smooth or rubber-tip forceps are preferred for smooth, friable foreign bodies, whereas alligator forceps are used for grasping sharp or irregular foreign bodies like plastic objects, coins, and bones [1]. Forceps are typically advanced to a few millimeters proximal to the foreign body. Then, the cups are opened maximally and advanced toward the foreign body under direct vision. The foreign body is gently but securely gripped and then extracted along with the bronchoscope as a unit. Sometimes, granulation tissue grows around the foreign body and may complicate the extraction. In that case, laser, argon plasma coagulation, or electrocautery can be used to release the foreign body [18]. Epinephrine can be used for hemostasis. Once the foreign body is removed, a repeat bronchoscopy should be done to check for another foreign body or residual parts. Glucocorticoids are occasionally used when a foreign body is surrounded by bleeding granulation tissue to decrease inflammation [22]. In those cases, glucocorticoids are administered for a few days before extraction. Antibiotics are used in case of clinical and/or radiological signs of infection [22]. If a patient develops stridor or subglottic edema after the foreign body extraction, intravenous glucocorticoids, aerosolized epinephrine, or helium-oxygen therapy may be considered [23].

Once foreign bodies are extracted, patients should be monitored for one to two days should complications arise. Complications such as noncardiogenic reexpansion pulmonary edema, airway inflammation, hemoptysis, pneumothorax, tracheoesophageal fistula, pneumonia, atelectasis, fever, and ventilatory failure have been reported [24]. If needed, a repeat flexible bronchoscopy should be considered a few days later.

Treatment of patients with symptoms of overdose from drug packet leak or rupture is supportive, including airway protection, respiratory and circulatory support, antiseizure drugs, and sometimes specific antidotes as needed. In cocaine toxicity, benzodiazepines should be used as soon as possible to reduce central nervous system sympathetic outflow [25]. Nitroprusside, nitroglycerin, or phentolamine may be used if blood pressure is not controlled with sedatives. Arrhythmias may be treated with sodium bicarbonate [26]. For patients requiring prolonged cardiopulmonary resuscitation after cardiac arrest or who develop heart failure, extracorporeal membrane oxygenation has been widely used as a bridge to recovery [27]. Also, therapeutic hypothermia helps to improve neurological outcomes in comatose patients after cardiac arrest [28].

## Conclusions

Clinicians should recognize the possibility of aspiration of illicit drug packets, especially in illicit drug users, as it can be life-threatening. Therefore, a high index of suspicion is crucial, and early recognition and prompt action can be lifesaving. In addition, physicians should be fully prepared regardless of the patient’s clinical status due to the possibility of sudden decompensation from foreign body dislodgement or rupture. Acute respiratory failure requires immediate airway management, followed by careful extraction of foreign body and treatment of any intoxication if present from absorption of the drug from the mucosa.
